# MM-3D Unet: development of a lightweight breast cancer tumor segmentation network utilizing multi-task and depthwise separable convolution

**DOI:** 10.3389/fonc.2025.1563959

**Published:** 2025-05-13

**Authors:** Xian Wang, Wenzhi Zeng, Junzeng Xu, Senhao Zhang, Yuexing Gu, Benhui Li, Xueyang Wang

**Affiliations:** ^1^ Attending Physician of Health Management Institute, The Second Medical Center and National Clinical Research Center for Geriatric Diseases, Chinese PLA General Hospital, Beijing, China; ^2^ Group of Agricultural High-Efficiency Water Management and Artificial Intelligence, College of Agricultural Science and Engineering, Hohai University, Nanjing, Jiangsu, China; ^3^ Department of Radiology and Nuclear Medicine, Xuanwu Hospital, Capital Medical University, Beijing, China; ^4^ Department of Cardiology, Yancheng Traditional Chinese Medicine Hospital Affiliated to Nanjing University of Chinese Medicine, Yancheng, Jiangsu, China; ^5^ Department of Radiology, Yancheng Traditional Chinese Medicine Hospital Affiliated to Nanjing University of Chinese Medicine, Yancheng, Jiangsu, China

**Keywords:** multi-task mobile 3D UNet, dynamic contrast enhanced MRI, breast cancer images segmentation, resource-constrained environments, convolutional neural networks

## Abstract

**Background and objectives:**

This paper introduces a novel lightweight MM-3DUNet (Multi-task Mobile 3D UNet) network designed for efficient and accurate segmentation of breast cancer tumors masses from MRI images, which leverages depth-wise separable convolutions, channel expansion units, and auxiliary classification tasks to enhance feature representation and computational efficiency.

**Methods:**

We propose a 3D depth-wise separable convolution, and construct channel expansional convolution (CEC) unit and inverted residual block (IRB) to reduce the parameter count and computational load, making the network more suitable for use in resource-constrained environments. In addition, an auxiliary classification task (ACT) is introduced in the proposed architecture to provide additional supervisory signals for the main task of segmentation. The network architecture features a contracting path for downsampling and an expanding path for precise localization, enhanced by skip connections that integrate multi-level semantic information.

**Results:**

The network was evaluated using a dataset of Dynamic Contrast Enhanced MRI (DCE-MRI) breast cancer images, and the results show that compared to the classical 3DU-Net, MM-3DUNet could significantly reduce model parameters by 63.16% and computational demands by 80.90%, while increasing segmentation accuracy by 1.30% in IoU (Intersection over Union).

**Conclusions:**

MM-3DUNet offers a substantial reduction in computational requirements of breast cancer mass segmentation network. This network not only enhances diagnostic precision but also supports deployment in diverse clinical settings, potentially improving early detection and treatment outcomes for breast cancer patients.

## 1 Introduction

Breast cancer has become the most common type of malignant tumor among women worldwide and remains a leading cause of cancer-related deaths among females (1). Surveys indicate that approximately 40,000 women die from breast cancer each year globally (2). Traditional breast cancer screening methods primarily rely on mammography, but they have limitations, particularly for dense breast tissue types more common in Asian populations. In recent years, advancements in magnetic resonance imaging (MRI) technology and techniques have propelled multimodal MRI scans to become the most sensitive imaging method for detecting breast cancer (3–5). This approach, demonstrating exceptional potential in the diagnosis of breast cancer, preoperative planning, and prognosis assessment, provides detailed anatomical images and accurately represents soft tissue structures (6). Among these modalities, Dynamic Contrast Enhanced MRI (DCE-MRI) plays a crucial role in the detection and diagnosis of breast cancer lesions (7). DCE-MRI sequences help identify significantly enhanced lesions and understand their size, signal, and morphological characteristics. These characteristics not only facilitate qualitative diagnosis based on lesion morphology and enhancement patterns, but also enable comparison between lesions before and after chemotherapy to evaluate treatment effectiveness (8, 9).

Traditional pathological tissue identification of breast cancer based on MRI imaging typically relies on the visual observation and subjective experience of physicians. However, However, the heterogeneity and atypical manifestations of breast cancer tumors, coupled with variations in physicians’ professional backgrounds, operational procedures, and working environments, pose a risk of misdiagnosis or overlooked cases. To address this, Computer-Aided Diagnosis (CAD) technology has emerged as a pivotal research area, augmenting doctors’ abilities in interpreting breast cancer pathological images (10). By enhancing diagnostic accuracy and consistency, while mitigating physicians’ workload and boosting efficiency, CAD technology significantly contributes to improved outcomes (11). One of hallmark features of breast cancer is the presence of a lump, underscoring the importance of accurately and efficiently detecting their size, shape, and margins in dense breast tissue. This has become a crucial challenge in CAD technology for breast cancer diagnosis (6). In recent years, the rapid developments of deep learning and computer vision technologies have led to significant achievements in Convolutional Neural Networks (CNNs) for semantic segmentation of breast cancer tumors (12, 13). By harnessing semantic features within images, CNNs merge pixels belonging to the same target class to create masks that highlight areas of interest, ultimately segmenting images into visually distinct regions with specific characteristics (14, 15). Deep learning algorithms leverage complex feature vector space computation framework and deeper computational layers to learn semantic features at various levels from intricate scene images without relying on manual feature extraction, thereby achieving more accurate end-to-end image semantic segmentation (16). The UNet network, characterized by its symmetrical encoder-decoder architecture with skip connections, demonstrates remarkable speed and accuracy in semantic segmentation, even when trained with limited datasets. Owing to its robust design and broad applicability, UNet has emerged as the preferred foundational framework for medical image segmentation models (17).

In the initial exploration of applying deep learning technologies to MRI image segmentation tasks, researchers attempted to decompose MRI images into multiple two-dimensional slices and subsequently applied 2D CNNs for semantic segmentation of these slices. The segmented results of each slice were then aggregated to produce the final segmentation output. For instance, Rachmadi et al. (18) employed transfer learning techniques to train UNet and UResNet networks, achieving commendable performance in the segmentation of brain tumor MRI images. Xiang et al. (19) proposed a 2D Dense-UNet network specifically designed for reconstructing high-quality MRI image sequences. 20) based on various 2D deep neural network (DNN) architectures, demonstrated the enhancement of brain tumor image segmentation through the use of multi-scale convolutions and cascaded structures.

Although 2D CNNs have achieved some success in MRI image segmentation, they often fail to consider the spatial continuity between slices, leading to the loss of crucial three-dimensional context information and relatively lower segmentation precision. Consequently, researchers have explored the integration of 3D CNN architectures that effectively capture spatial information in MRI image segmentation. Dong et al., introduced a symmetrically structured 3DUNet model that excelled in segmenting breast cancer tumor MRI images (21). Inspired by the 2D structure of UNet. Milletari et al. (22) developed VNet, a 3D CNN that enhanced three-dimensional image segmentation accuracy through improvements in network sampling layers and loss functions. Islam et al. (23) fused channel and spatial attention mechanisms with the 3D UNet architecture to create an MRI image segmentation model that surpassed previous efforts in terms of performance. Subsequently, numerous researchers proposed diverse modifications to the 3D UNet by incorporating intricate network architectures, dimension fusion strategies, and rich auxiliary modules, which significantly improved medical image segmentation accuracy but also increasing model complexity and computational demands (24). While these enhancements are beneficial, they come at the expense of increased parameter count and training costs which pose challenges for practical deployments, particularly in resource-constrained settings such as mobile health devices and edge computing platforms. Thus, there is a pressing need for lightweight strategies that strike a balance between accuracy and efficiency to meet diverse medical image segmentation needs.

This paper introduces a novel 3D U-Net architecture, termed MM-3DUNet, tailored specifically for the seamless and efficient segmentation of intricate breast cancer tumors in clinical settings. Built upon continuous three-dimensional DCE-MRI images, MM-3DUNet offers a lightweight solution for semantic segmentation. At its core, the network employs 3D separable convolutions, which serve as the backbone for enhancing feature representation during information propagation. It incorporates innovative design elements such as channel expansion convolution unit and inverted residual blocks, further bolstering its feature extraction capabilities. By adeptly processing breast MRI data, MM-3DUNet achieves precise and accurate segmentation of complex breast cancer tumors, demonstrating its potential to streamline clinical workflows and improve diagnostic accuracy.

## 2 Methods

The classical 3DU-Net captures rich contextual information in three-dimensional space, enabling precise segmentation of complex tumors from consecutive breast cancer MRI images. However, its practical application is hindered by substantial model parameter size, high computational demand, and significant memory consumption, which limits its deployment in resource-constrained environments. Therefore, this paper introduces a lightweight semantic segmentation MM-3DUNet network based on 3D separable convolutions. This network enhances feature expression during information flow through channel expansion convolution unit and inverted residual blocks. Additionally, auxiliary classification task provides valuable supervisory signals for the primary task of breast cancer tumor segmentation. MM-3DUNet maintains segmentation accuracy while significantly reducing model parameters and computational load.

### 2.1 The network architecture of MM-3DUNet

**Figure 1:**
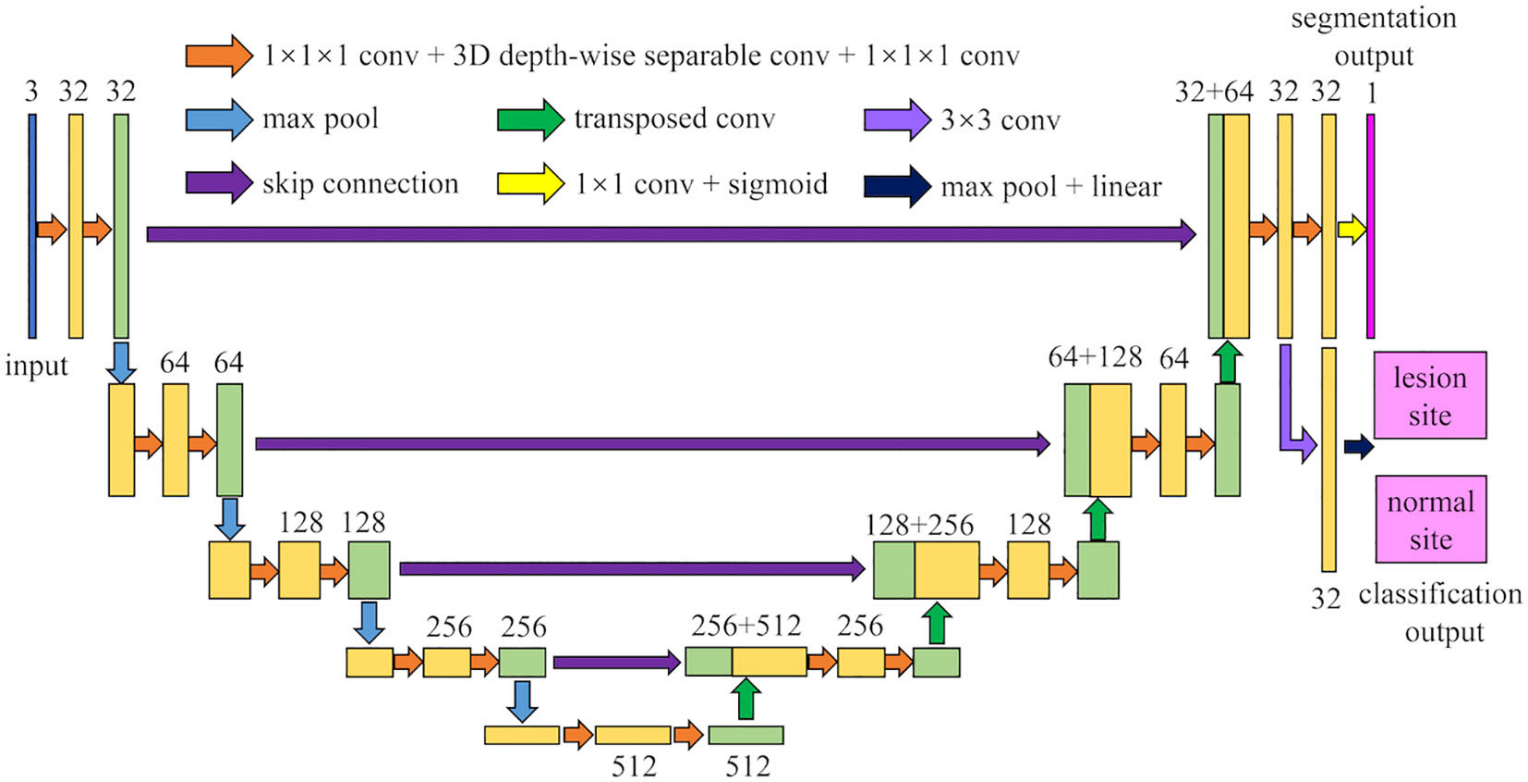
The network architecture of MM-3DUNet.

The architecture of MM-3DUNet (Multi-task Mobile 3D UNet) is depicted in **Figure 1**. Similar to the classical 3DU-Net, MM-3DUNet consists of a progressively contracting downsampling pathway and a symmetrically expanding upsampling pathway, constituting the encoder and decoder of the network, respectively. The encoder extracts multi-level semantic features from the input image and compresses the feature map dimensions through four successive computational modules, each containing two repeated unit of feature extraction. Each unit comprises two convolutions with kernel size of 1×1×1 and one depth-wise separable convolution, followed by activation functions. A max pooling layer at the end of the second feature extraction unit reduces the feature map size. Each computational module doubles the number of image channels while halving their dimensions. Conversely, the decoder restores both feature map size and localizes critical information to generate a target image of the same size as the original image. It similarly consists of computational modules with two repeated feature recovery unit, where each unit concludes with a transpose convolution to expand feature map size. After feature recovery, each module halves the number of image channels while doubling their dimensions again. Skip connections between corresponding layers in both encoder and decoder facilitate integration between low-level and high-level semantic information effectively. The network concludes with a 1×1×1 convolution to reduce the final feature map’s channel count to the number of labels, subsequently, predictions are transformed into probability values ranging from 0 to 1 using sigmoid function activation. Additionally, an auxiliary classification task at the end of encoder differentiates between normal and lesion areas using a classifier composed of a 3×3 convolution layer followed by max pooling layer and linear activation layer.

### 2.2 3D depth-wise separable convolution

**Figure 2:**
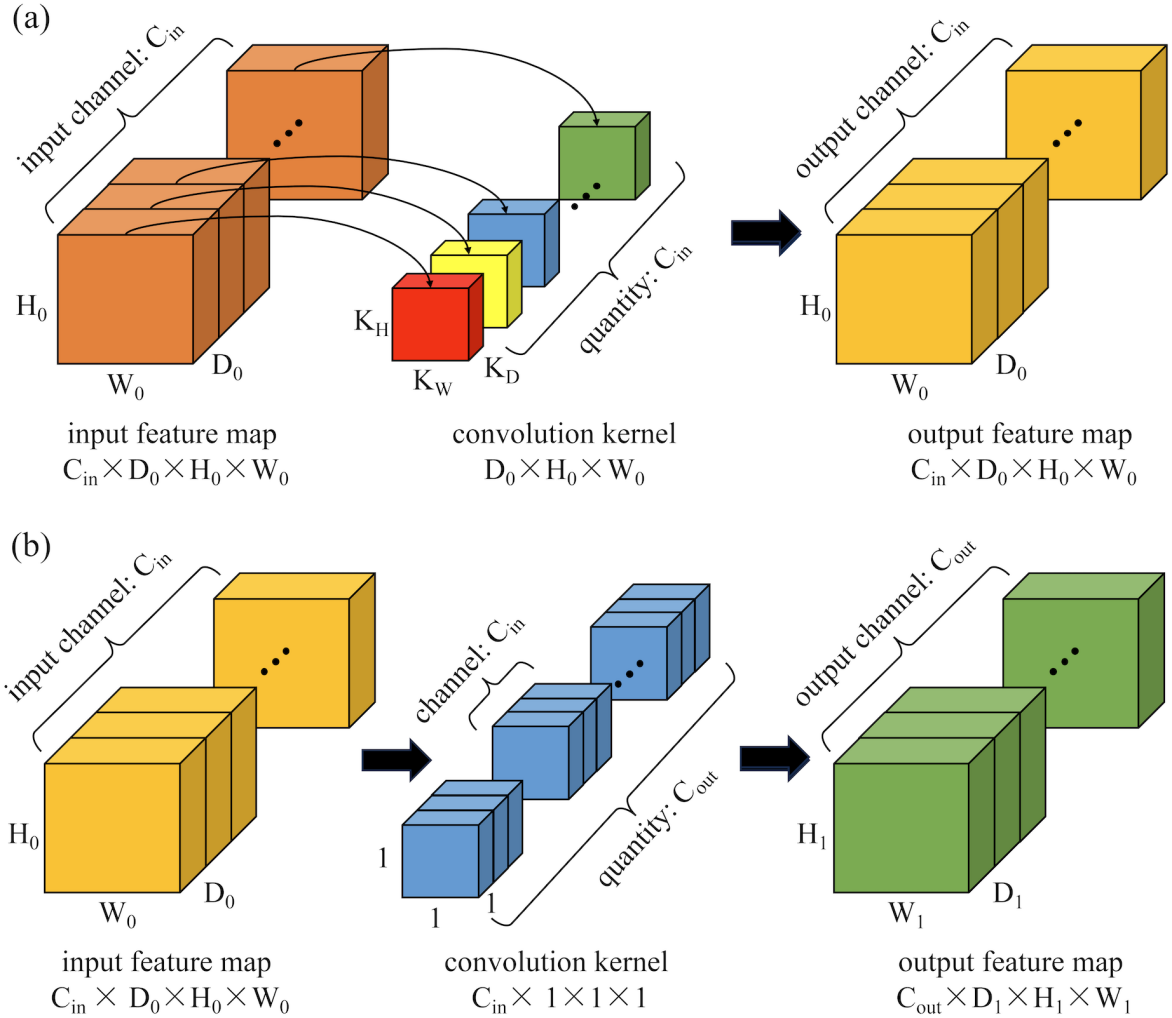
Schematic diagram of the computation process for 3D depth-wise separable convolutions: **(a)** 3D depth convolution; **(b)** 3D pointwise convolution.

Inspired by the concept of depth-wise separable convolutions in the Xception model (25), this paper proposes a 3D depth-wise separable convolution to replace the conventional 3D convolutions in the classic 3DU-Net. This replacement effectively reduces the parameter count and computational complexity of the model. The proposed 3D depth-wise separable convolution is divided into two primary steps: 3D depth-wise convolution and 3D pointwise convolution. Unlike standard 3D convolutions that combine all input channels with each convolution kernel, convolution operations are performed independently on each input channel in 3D depth-wise convolution. This process is illustrated in **Figure 2a**. Assuming the input feature map has C_in_ channels with dimensions D_0_×H_0_×W_0_ (where D_0_, H_0_, and W_0_ represent the depth, height, and width of the input features, respectively), C_in_ kernels of size K_D_×K_H_×K_W_ are used to separately convolve with each channel, resulting in an output feature map still having Cin channels. The second step is a 3D pointwise convolution depicted in **Figure 2b**, which is similar to standard convolution operations but uses C_out_ 1x1x1 kernels with C_in_ channels to combine outputs from the previous step along the channel dimension. Ultimately, this produces an output feature map with C_out_ channels and dimensions D_1_×H_1_×W_1_.

The parameter count (P_3D-ds_) and computational load (F_3D-ds_) of the entire 3D depth-wise separable convolution process are calculated as follows:


(1) $$P_{3D-ds}=C_{in}\times K_{D}\times K_{H}\times K_{W}+C_{in}\times C_{out}$$

(2) $$F_{3D-ds}=D\times H\times W\times C_{in}\times (K_{D}\times K_{H}\times K_{W}+C_{out})$$

If standard 3D convolution operations are used to achieve the same feature extraction effect, the parameter count P_3D-standard_ and computational load F_3D-standard_ of the network would be calculated as follows:


(3) $$P_{3D-standard}=C_{in}\times K_{D}\times K_{H}\times K_{W}\times C_{out}$$

(4) $$F_{3D-standard}=D\times H\times W\times C_{in}\times K_{D}\times K_{H}\times K_{W}\times C_{out}$$

To compare the parameter count and computational load of the proposed 3D depth-wise separable convolution with those of standard 3D convolution.


(5) $$\begin{array}{c} \frac{P_{3D-ds}}{P_{3D-standard}}=\frac{C_{in}\times K_{D}\times K_{H}\times K_{W}+C_{in}\times C_{out}}{C_{in}\times K_{D}\times K_{H}\times K_{W}\times C_{out}}\\ =\frac{1}{C_{out}}+\frac{1}{K_{D}\times K_{H}\times K_{W}} \end{array}$$

(6) $$\begin{array}{c} \frac{F_{3D-ds}}{F_{3D-standard}}=\frac{D\times H\times W\times C_{in}\times (K_{D}\times K_{H}\times K_{W}+C_{out})}{D\times H\times W\times C_{in}\times K_{D}\times K_{H}\times K_{W}\times C_{out}}\\ =\frac{1}{C_{out}}+\frac{1}{K_{D}\times K_{H}\times K_{W}} \end{array}$$

It can be observed that after employing 3D depth-wise separable convolutions, both the parameter count and computational load are only $\frac{1}{C_{out}}+\frac{1}{K_{D}\times K_{H}\times K_{W}}$ of their original values. This significantly minimizes the model’s parameter count and computational complexity.

### 2.3 Channel expansional convolution

**Figure 3:**
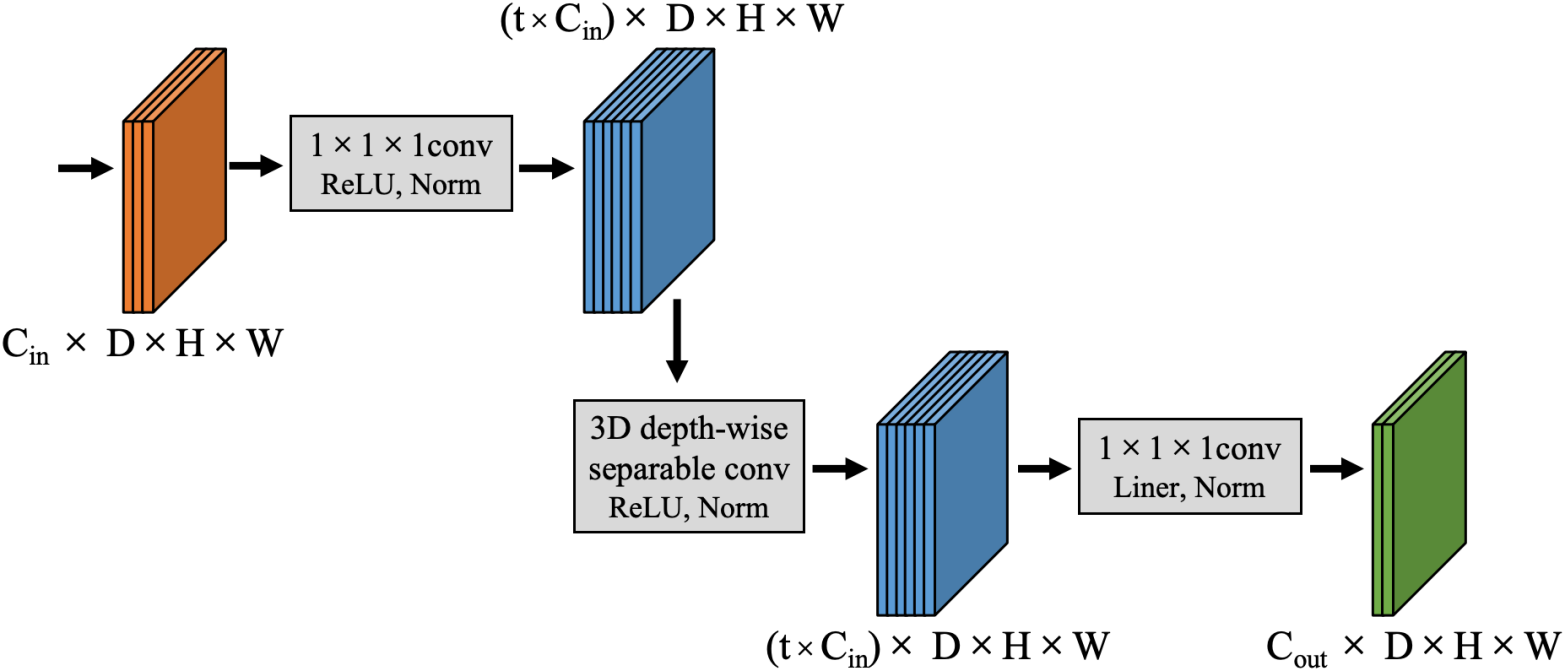
The architecture of Channel Expansional Convolution.

Compared to low-dimensional spaces, computations in high-dimensional spaces facilitate more complex feature learning patterns. This allows models to uncover latent information within the data, which is challenging to capture through low-dimensional features, providing a more comprehensive description of data characteristics. Consequently, deep learning models can discern subtler and more intricate relationships and patterns within the data. To this end, this study introduces a CEC unit which initially expands the dimensionality of input features to map them from low-dimensional inputs to a high-dimensional space for enhanced feature extraction. Subsequently, it reduces the dimensionality of features to alleviate the computational burden on subsequent layers, thereby boosting model efficiency without significantly increasing computational load or parameter count. This proposed CEC unit enhances network expressiveness and nonlinearity without substantial increases in computational demand. The structure of the CEC unit is depicted in **Figure 3**, primarily consisting of three stages: channel expansion, depth-wise convolution, and channel compression.

During the channel expansion phase, for input features of size C_in_×D×H×W, a 1×1×1 convolutional kernel first expands the channel dimension to t×C_in_, where t is the channel expansion factor that regulates the extent of channel expansion. This produces a feature map with enriched dimensional information, sized (t×C_in_)×D×H×W. Subsequently, in the depth-wise convolution phase, a 3×3×3 3D depth-wise separable convolution is employed to independently extract features from each channel, thereby reducing the model’s computational load while maintaining stable internal spatial structure. The output feature map dimensions remain (t×C_in_)×D×H×W. Finally, during the channel compression stage, another 1×1×1 convolutional kernel reduces the channel count from t×C_in_ to C_out,_ significantly decreasing both the computational load and the number of parameters. Notably, after each convolution in the channel expansion and depth-wise convolution stages, a combination of Leaky ReLU activation function and Instance Norm normalization is used for nonlinear transformation and channel normalization of the feature map which enhances nonlinearity and stability in feature extraction. During the channel compression stage, a linear activation function is employed after convolution in the low-dimensional space to perform linear transformation of the feature map so as to avoid loss of nonlinear information while preserving more original image information.

### 2.4 Inverted residual block

**Figure 4:**
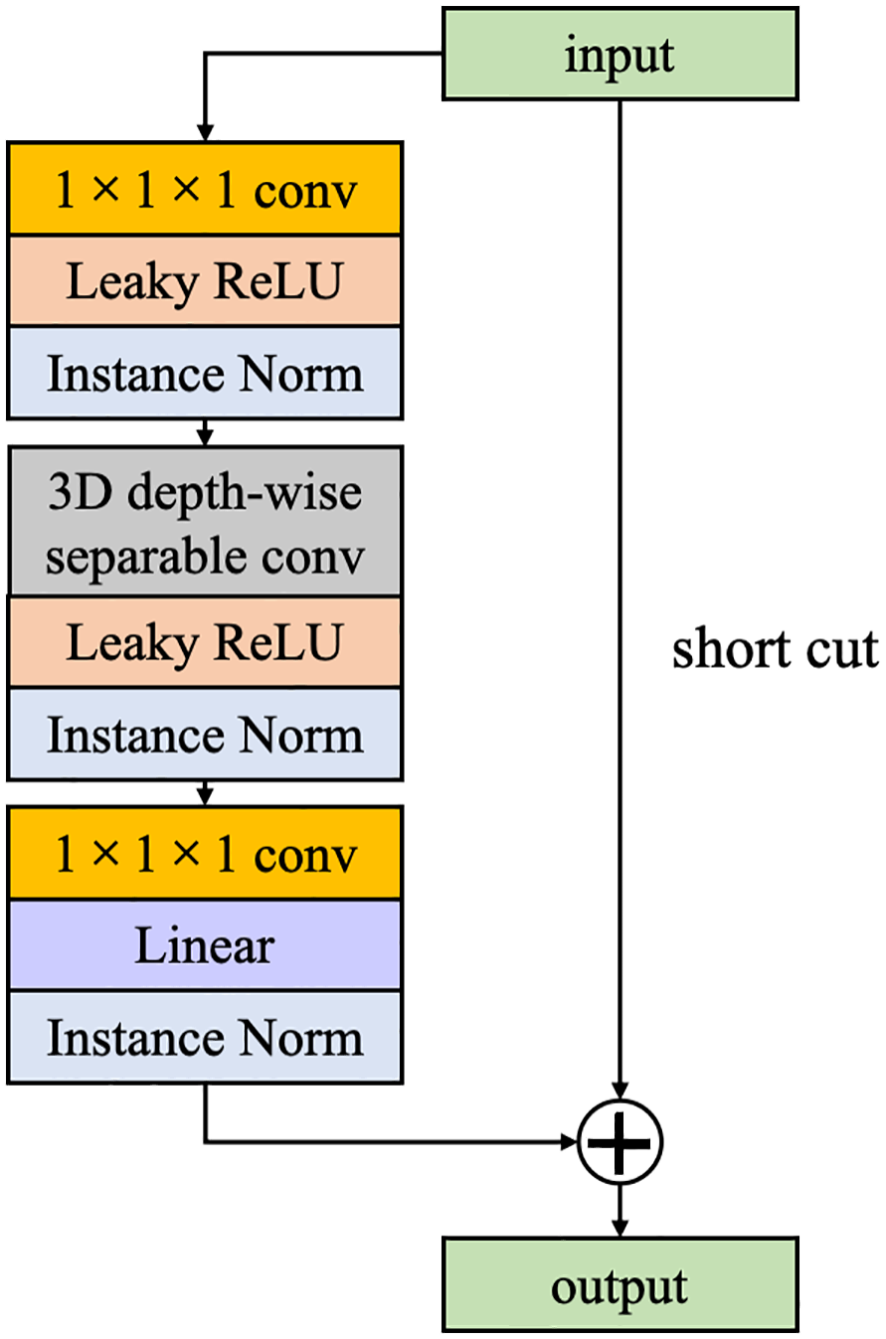
The architecture of Inverted Residual Block (IRB).

Inspired by ResNet, we have incorporated IRB into the CEC unit. Unlike traditional residual blocks where an identity mapping is added to connect the input features to the output while the number of channels remains constant. In this study, the opposite strategy is used, where the channel of input features is first expanded, and the features are extracted and then compressed. If both input and output features pose identical channel numbers, a direct addition of the input feature map to the output feature map forms a residual connection. This strategy not only enhances the efficiency of information transfer across the computational unit but also preserves part of the input features, effectively mitigating the issue of gradient vanishing and enhancing feature representational capacity (**Figure 4**).

### 2.5 Auxiliary classification task

**Figure 5:**
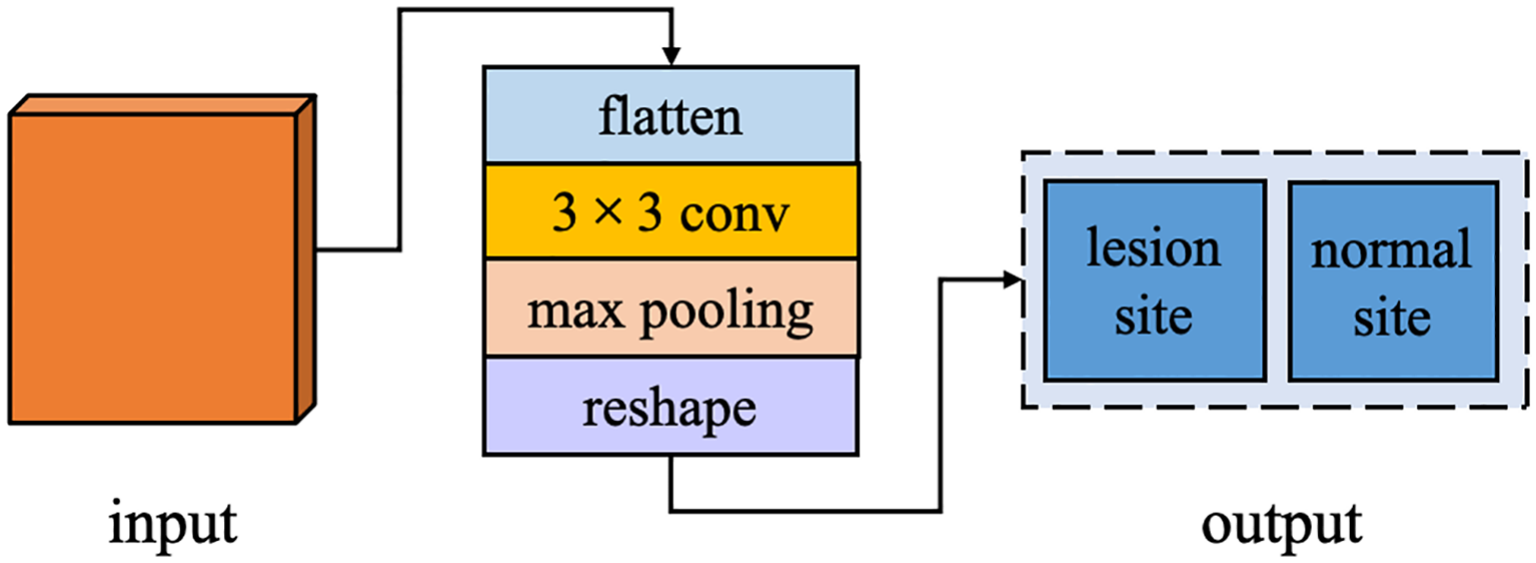
The architecture of ACT.

In the task of breast cancer tumor segmentation, leveraging the global feature differences between lesion and normal regions can enhance the accuracy and stability of segmentation. Therefore, we have augmented the decoder stage of our network with an ACT branch, designed to determine whether the input image contains a lesion site. This branch operates in parallel with the primary task of breast cancer tumor segmentation within the overall deep learning framework. ACT bypasses the correlation between breast cancer lesion segmentation and lesion and normal tissue classification by sharing the image features extracted from the shared encoding stage and adds additional supervisory signals from the perspective of learning global feature information. This approach supplies the network decoder with more global contextual information, fostering the learning of more generalized feature representations, preventing overfitting in the main task, and improving the performance of the semantic segmentation task. The structure of ACT is illustrated in **Figure 5**, including an additional parallel classifier unit into the final computation module of the encoder. After feature map restoration, it first maps these features to a two-dimensional space, then employs a 3×3 convolutional kernel to extract type features from the input image, followed by a pooling layer to adjust the data structure, and finally, a linear activation layer outputs results corresponding to the number of types.

### 2.6 Data processing

#### 2.6.1 Breast MRI image acquisition

In this study, we acquired multimodal MRI data for breast cancer using a Discovery MR 750w system from GE Healthcare, which was equipped with an eight-channel dedicated breast phased-array coil. Patients were positioned in the prone position with breasts naturally pendant within the coil cavity. Scanning parameters and sequences followed standard protocols, including non-contrast and dynamic contrast-enhanced scans. Specific parameters for some of the non-contrast sequences included a VIBRANT sequence with a TR of 7.6 ms, TE of 4.2 ms, a matrix of 256×256, a slice thickness of 1.00 mm, and no interslice gap. The contrast agent used was gadopentetate dimeglumine (GD-DTPA; Magnevist^®^; Bayer, Berlin, Germany), at a dose of 0.1 mmol/kg administered via antecubital vein at a flow rate of 2 ml/s. Imaging was performed before contrast injection (mask phase) and continued for six subsequent acquisitions post-injection, totaling seven phases in total duration. These configuration parameters were carefully selected to ensure optimal clarity and resolution of the data to facilitate subsequent image segmentation and analysis tasks.

#### 2.6.2 Dataset construction

**Figure 6:**
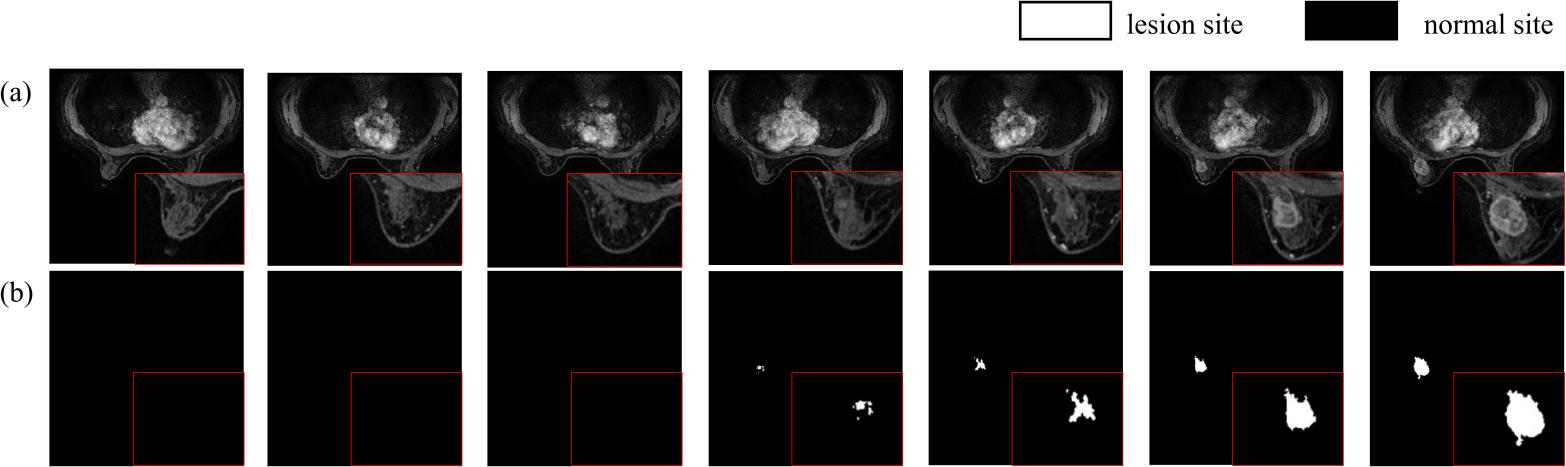
Breast Cancer Tissue Labels: **(a)** Breast Tissue; **(b)** Breast Cancer Tumor Labels.

In this study, the data were diagnosed by two experienced radiologists with over ten years of expertise. Lesion delineation was performed using a dedicated GE workstation to draw time-signal enhancement curves from dynamic contrast-enhanced MRI scans, specifically selecting the phase where lesions showed maximum enhancement in VIBRANT images. The original DICOM images were converted to NIfTI format, and lesion delineation was conducted utilizing ITK-SNAP software. This process was further refined by incorporating ADC values from DWI sequences to ensure the accuracy of lesion marking, ultimately producing labeled images of breast cancer tumors (**Figure 6**). The dataset comprises 219 breast tissue samples, each containing 184 MRI slices, resulting in a total of 40,296 images. After converting DICOM images to NIfTI format, comprehensive preprocessing was applied: (1) Intensity normalization using Z-score standardization across all MRI sequences to mitigate scanner variability; (2) Rigid spatial alignment of DCE-MRI phases via Elastix toolkit to correct patient motion artifacts; (3) Random volumetric augmentation including ±15° rotation along axial plane, horizontal flipping (50% probability), and ±20% linear intensity scaling to improve model robustness. The original dataset was divided into training and test sets at an 8:2 ratio. During training, consecutive sets of 16 or 32 slices were randomly selected as input for the model.

#### 2.6.3 Experimental environment configuration

This study was conducted using the Ubuntu 20.04 LTS operating system for development and testing, employing Python 3.8 as the programming language. All model training, validation, and testing processes were performed under the deep learning framework Pytorch v1.13. The computational hardware included a 13th Gen Intel(R) Core(TM) i5-13600K CPU, 64GB of RAM, and an NVIDIA GeForce RTX 3090 GPU. Hardware acceleration was facilitated by leveraging the CUDA 12.2 computing platform along with the cuDNN 8.9.1 library optimized for deep learning operations.

### 2.7 Model parameter configuration

During network training, the number of epochs was set to 130 with a batch size of 24. The Adam optimizer was employed with a weight decay rate of 0.0001 and a momentum factor of 0.95. Dice Loss was utilized as the loss function for segmentation tasks, while ACT were addressed using Cross-Entropy Loss (CE Loss). Dice Loss aggregates all pixels of a category to compute the loss, directly using the segmentation performance metric as a supervisory signal. This approach disregards a significant amount of background pixels in the intersection-over-union calculation, effectively addressing the issue of class imbalance and facilitating faster convergence. The formulations for Dice Loss and CE Loss are as follows:


(7) $$L_{dice}=1-\frac{2I+\epsilon }{U+\epsilon }=1-\frac{2\sum_{i=1}^{H\times W}p(c_{i})g(c_{i})+\epsilon }{\sum_{i=1}^{H\times W}p(c_{i})+\sum_{i=1}^{H\times W}g(c_{i})+\epsilon }$$

(8) $$L_{ce}=-\sum_{i=1}^{H\times W}g(c_{i})\text{log }p\;(c_{i})$$

In the formula, p(c_i_) represents the label value of the i-th pixel for class C, which can take values of either 0 or 1. Meanwhile, g(c_i_) denotes the predicted probability that the i-th pixel belongs to class C. I is the intersection of the predicted and label values; H and W are the height and width of the feature map, respectively; U is the union of the predicted and label values; whereas ϵ is a smoothing coefficient introduced to prevent division by zero, typically set to a very small positive number. Additionally, the network training process employs a Cosine Annealing Learning Rate as the rate decay strategy, with its calculation formula presented as follows:


(9) $$lr_{t}=\frac{lr_{0}}{2}\left[1+\cos\left(\frac{t\pi }{T}\right)\right]$$

In the equation, lr_t_ represents the learning rate for each training iteration; lr_0_ is the initial learning rate, set to 0.0001; T denotes the number of training iterations in one cosine cycle, which is equivalent to the number of epochs in the network.

### 2.8 Evaluation metrics

In image semantic segmentation tasks, evaluating the performance of deep learning networks comprehensively requires not only assessing the accuracy of individual target pixel classification but also comparing the overall segmentation results with the segmentation labels. Therefore, this study employs the Intersection over Union (IoU) as an evaluation metric for evaluating the accuracy of the MM-3DUNet model. IoU quantifies the ratio of the intersection to the union between the predicted target area pixels and the actual annotated target area pixels, reflecting the overlap degree between the segmentation results and the annotated labels. The calculation formula is as follows:


(10) $$IoU=\frac{TP}{TP+FN+FP}$$

where TP represents the number of pixels accurately identified as the target category, FP denotes the pixels incorrectly classified as the target category despite being labeled as non-target, TN refers to the pixels correctly identified as non-target, and FN encompasses the pixels erroneously classified as non-target despite being labeled as the target category.

In parallel, this study also adopts the Dice Similarity Coefficient (DSC) as a complementary evaluation metric to assess segmentation accuracy. The DSC measures the spatial overlap between the predicted segmentation and the ground truth annotations, emphasizing the consistency of positive classifications. It is calculated by harmonizing the ratio of twice the intersection area to the sum of the predicted and annotated target areas, thereby providing sensitivity to both over- and under-segmentation errors. The mathematical formulation is defined as:


(11) $$DSC=\frac{2\times TP}{2\times TP+FN+FP}$$

The DSC ranges from 0 to 1, with higher values reflecting greater congruence between algorithmic outputs and expert annotations, particularly in scenarios with class imbalance or irregular lesion morphology.

Additionally, Floating Point Operations (FLOPs) and the total number of Parameters were used as evaluation metrics in order to evaluate the changes in computational complexity of the improved segmentation network. FLOPs represent the number of floating-point operations required for one forward pass of the model, directly reflecting the computational workload. A higher FLOP count indicates an increased computational cost and extended processing time due to more floating-point calculations performed during computation. Parameters denote the total count of all model components, including weights and biases, influencing not only the computational complexity but also impacting the data volume requirements and training duration. Excessive parameters can lead to model overfitting, while insufficient parameters may limit the model’s representational capacity.

## 3 Results

### 3.1 Model performance validation and comparison

**Figure 7:**
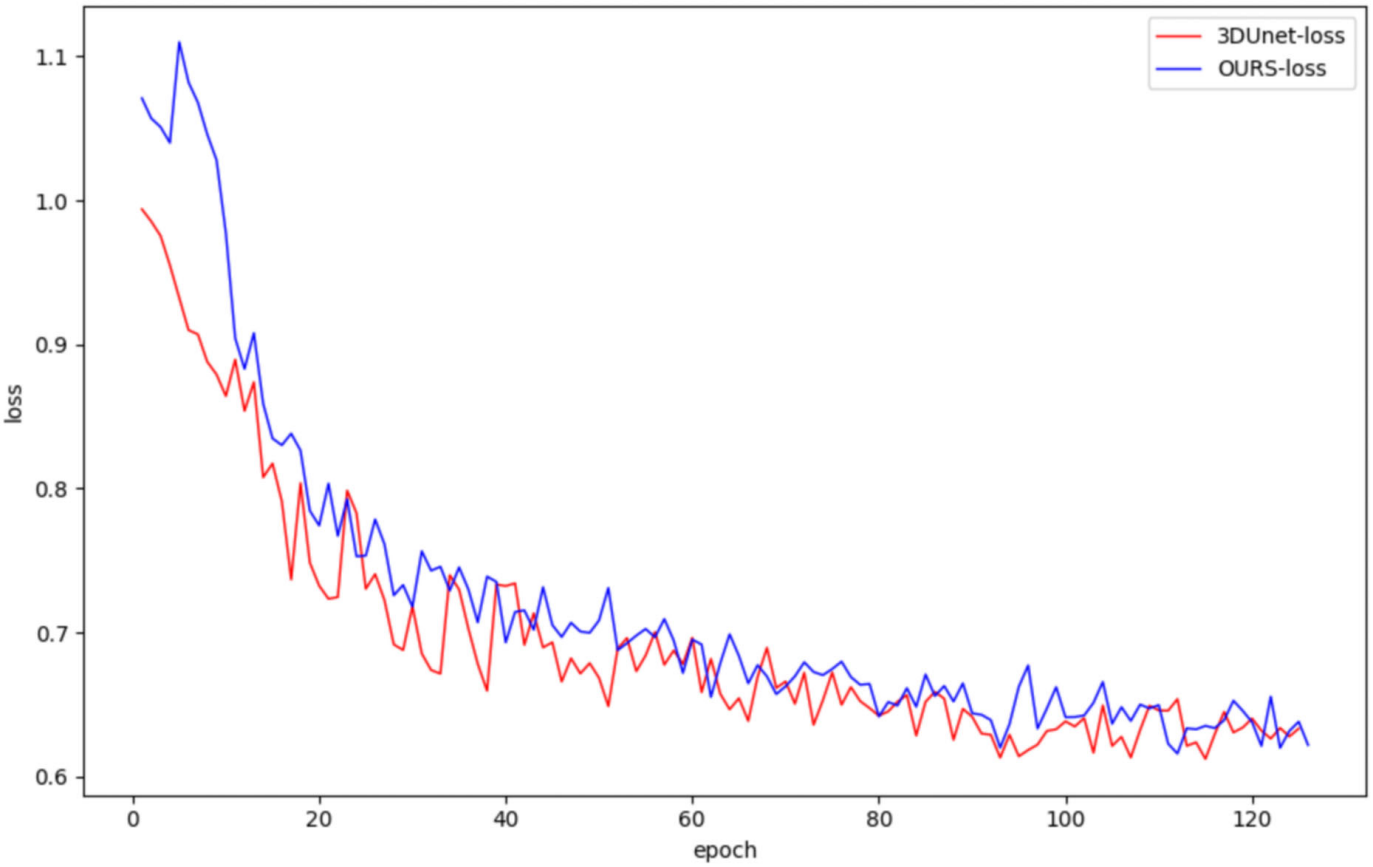
Comparison of loss value change curve between 3DUNet and MM-3DUNet in verification.

**Figure 8:**
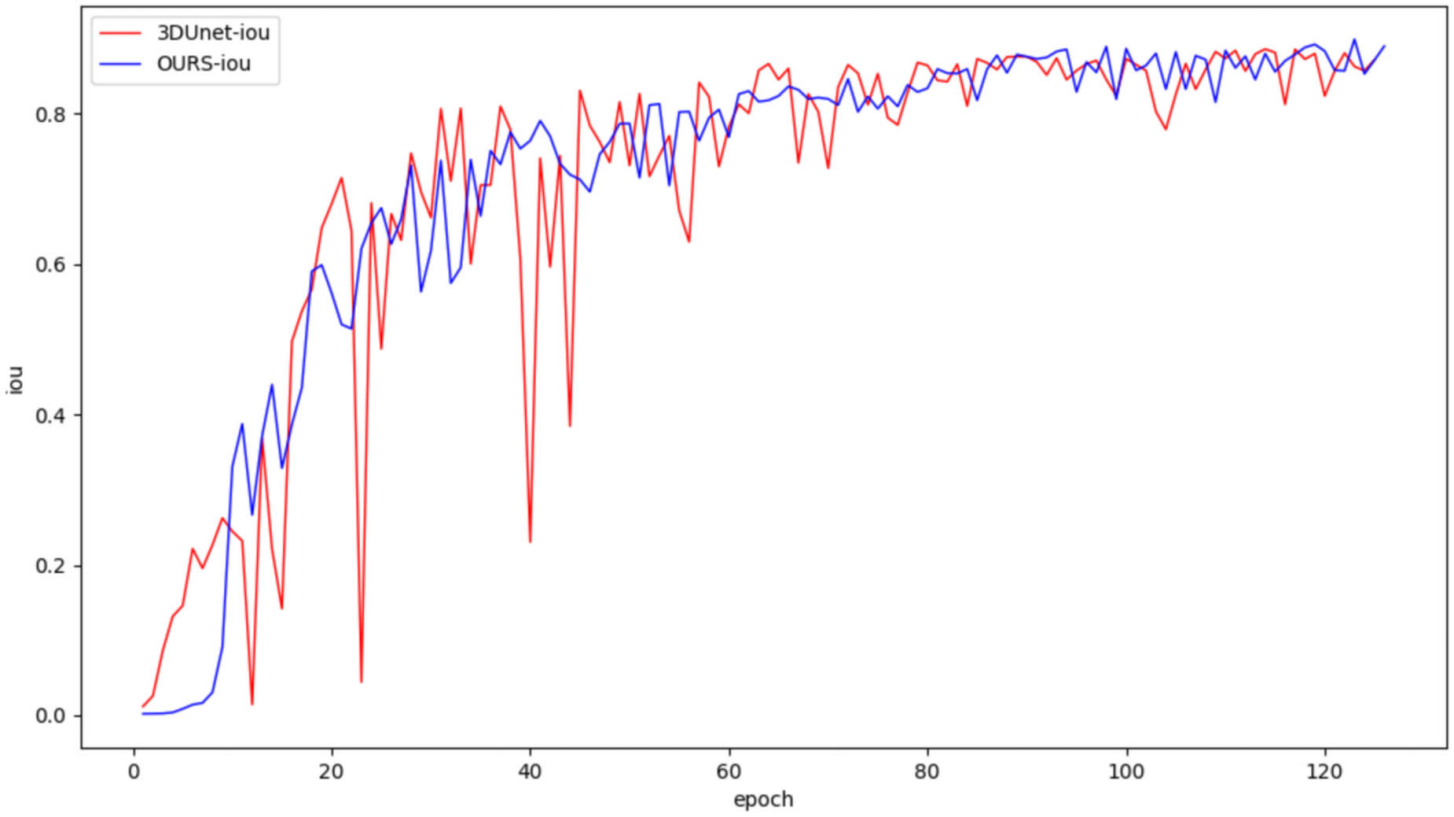
Comparison of IoU change curves between 3DUNet and MM-3DUNet in verification.

In this study, both the classical 3DUNet and the proposed lightweight 3D semantic segmentation network, MM-3DUNet, were trained using the same breast cancer pathology image dataset and model parameter settings to validate the superior performance and computational efficiency of MM-3DUNet. The changes in the loss function and Intersection over Union (IoU) for both models during validation are illustrated in **Figures 7** and **8**, respectively. As depicted in **Figure 7**, the loss values for both 3DUNet and MM-3DUNet decrease progressively with each epoch. Notably, our MM-3DUNet demonstrates a more rapid reduction in loss value, indicating enhanced learning efficiency that enables swift feature extraction from data and effectively parameters adjustment for thorough information extraction. Comparison of the IoU curves in **Figure 8** reveals a gradual increase in IoU over epochs for both models, however, MM-3DUNet exhibits a significantly faster rate of improvement. This further confirms the efficiency of MM-3DUNet during training. Additionally, smaller fluctuations in the IoU curve of MM-3DUNet suggest its enhanced stability and reduced susceptibility to variations or noise in the training data, demonstrating superior performance and reliability in the task of breast cancer tumor segmentation.

### 3.2 Performance of 3D depth-wise separable convolutions

To validate the effectiveness of the proposed 3D depth-wise separable convolutions in reducing the number of network parameters and computational complexity, we employed the classical 3DUNet as a baseline model with standard 3D convolutions. We replaced the convolutional unit in this baseline with 3D depth-wise separable convolutions and conducted training and evaluation on the same dataset. The accuracy and computational complexity of both networks were compared using the test set, with results presented in **Table 1**. It shows that replacing standard 3D convolutions with 3D depth-wise separable convolutions significantly reduced the computational complexity of the breast cancer tumor segmentation network. Specifically, the number of parameters decreased by 1.57M to 11.8% of the baseline, while FLOPs were reduced by 88.05G to 23.5% of the baseline. Moreover, employing these new convolutions resulted in nearly unchanged IoU and DSM, with only a minor reduction of 0.97% and 0.55%, maintaining high segmentation accuracy.

**Table 1:** Comparison of breast cancer tumor segmentation performance between different models.

Model	Expansion factor	Parameters (M)	FLOPs (G)	IoU (%)	DSC(%)
3DUNet	–	1.78	115.10	88.61	93.96
MM-3DUNet	6	0.21	27.05	87.64	93.41
MM-3DUNet	12	0.33	41.79	89.10	94.24
MM-3DUNet+ACT	12	0.34	42.40	89.91	94.69

These results demonstrate that the proposed 3D depth-wise separable convolutions can efficiently adapt to the complex structural characteristics of three-dimensional data, thereby capturing both spatial and depth correlations. Furthermore, while maintaining comparable model performance, this approach significantly reduces the number of floating-point operations required during forward propagation and the overall parameter count. Consequently, it enables faster data processing during training and inference, achieving enhanced computational efficiency and reduced resource consumption. As a result, this methodology facilitates the deployment of models on resource-constrained devices such as mobile and embedded systems.

### 3.3 Performance of channel expansion convolution unit

Based on 3D depth-separable convolution, a channel expansion convolution unit is designed to improve the accuracy of breast cancer tumor feature extraction. In order to explore the effectiveness of this structure, a comparative experiment was designed as shown in **Table 1**. By setting different channel expansion factors, adjusting the number of channels in the network expansion layer and changing the diversity of feature learning, we compared the performance of different networks on the test set. The results demonstrate that small channel expansion factor leads to a significant decrease in network computing complexity but compromises segmentation accuracy compared with the Baseline. Notably, when adjusting MM-3DUNet’s channel expansion factor from 6 to 12, IoU increased by 1.46%, DSM increased by 0.83%, surpassing classic 3DUNet by 0.49%. From the aspect of network computing complexity, the Parameters of MM-3DUNet decreased by 1.45M and FLOPs decreased by 73.31G compared with Baseline.

**Figure 9:**
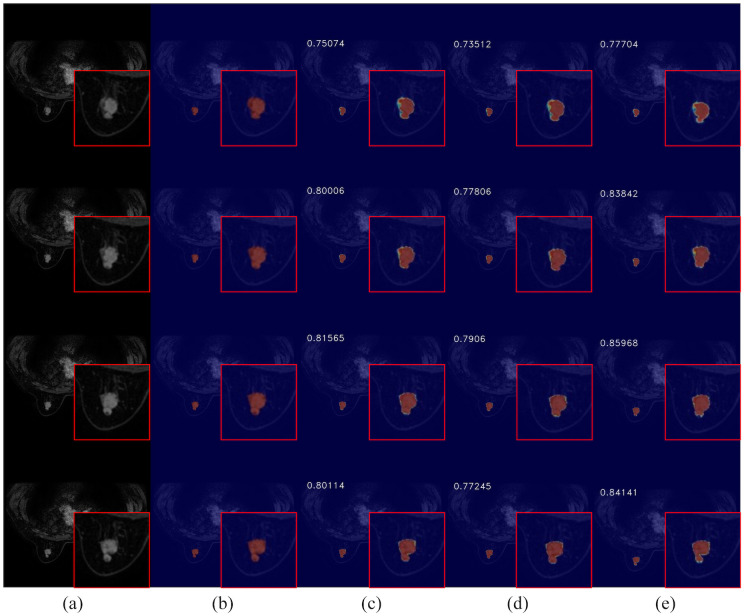
Comparison of breast cancer mass segmentation accuracy before and after using a CEC unit network on the test set: **(a)** breast tissue section; **(b)** breast cancer mass labeling; **(c)** segmentation results from the 3DUNet network; **(d)** segmentation results from the MM-3DUNet network with an expansion factor of 6; **(e)** segmentation results from the MM-3DUNet network with an expansion factor of 12. (The IoU value is displayed in the upper left corner of each segmentation effect diagram.).

To further evaluate the impact of CEC unit on segmentation performance, we compared the visual results of breast cancer mass segmentation accuracy among different networks in the test set, as shown in **Figure 9**. As illustrated in the figure, directly adding channel expansion convolution leads to a decrease in the segmentation accuracy of the network. Compared to 3DUNet, there is a greater absence of edge details and insufficient recognition accuracy for fuzzy regions in these segmentation results. However, by appropriately adjusting the channel expansion factor, significant improvement was observed in the segmentation precision of the network for complex areas within image compared to Baseline, resulting in more refined contour restoration for breast cancer lump region.

The experimental results show that CEC unit effectively adjust the complexity and feature extraction capability of the model while aiding removal of redundant information and retention of key features, so as to obtain a more compact and effective feature representation which balance the representation capability and computational complexity of the model. Furthermore, when combined with 3D depth-separable convolution, it reduces computational load on network while enhancing segmentation accuracy.

### 3.4 Performance of ACT

On the basis of the existing MM-3DUNet, we further incorporated the ACT. Following training and verification on the same dataset, we compared the performance of different networks on the test set, as shown in **Table 1**. The results demonstrate that with the addition of ACT, the IoU and DSM of MM-3DUNet increases by 1.30% and 0.73% compared with those of 3DUNet, while reducing Parameters and FLOPs by 63.16% and 80.90% respectively. The results highlight that incorporating an ACT provides additional supervisory signals about breast cancer lesions within the network architecture, facilitating learning of more generalized feature representations, and preventing overfitting through appropriate network complexity augmentation, thus enhancing the segmentation performance of the main task.

**Figure 10:**
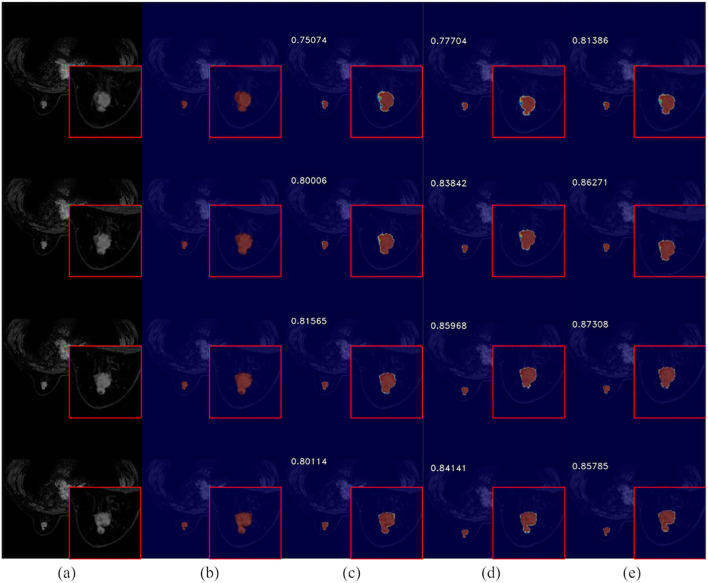
Comparison of breast cancer mass segmentation accuracy on a test set before and after the incorporation of ACT: **(a)** breast tissue sections; **(b)** labeled breast cancer masses; **(c)** segmentation results from the 3DUNet network; **(d)** segmentation results from the MM-3DUNet network; **(e)** segmentation results from the MM-3DUNet+ACT network. (The Intersection over Union (IoU) values are displayed in the upper left corner of each segmentation result image.).

The integration of ACT into the network is illustrated in **Figure 10**, showcasing its ability to enhance segmentation visual effects. This addition notably refines the boundary segmentation of breast cancer tumors, resulting in a closer alignment with the actual conditions. Consequently, it aids in a more accurate understanding of spatial relationships between the target region and surrounding pixels, thereby enabling precise delineation of pathological site boundaries by the network. Furthermore, the augmented network exhibits improved performance in handling complex cases as suggested by enhanced visual effects. It effectively addresses challenges such as variability in mass shape and indistinct boundaries, thereby providing a more dependable foundation for early diagnosis and treatment of breast cancer.

## 4 Discussion

In this study, we developed MM-3DUNet, a lightweight breast cancer mass segmentation network which integrates depth-wise separable convolutions with 3D convolutional neural networks. To further enhance its performance, we introduce CEC unit and an IRB to replace traditional simple 3D convolutional unit. This novel design successfully focuses computational resources on more representative features, reducing redundant calculations, thereby maintaining or even improving segmentation accuracy while significantly lowering the model’s parameter count and computational demands. Specifically, by decomposing standard 3D convolutions into depth-wise and pointwise convolutions, our approach of using 3D depth-wise separable convolutions effectively reduces the network’s parameter count and computational load without compromising feature extraction capabilities.

Previous studies primarily relied on traditional manually extracted MRI features for distinguishing benign from malignant breast cancer tumors and predicting breast cancer molecular subtypes. Although recent research have adopted semi-automatic radiomics approaches for feature extraction, these methods still depend on manual assessment of critical lesion characteristics (26, 27). Particularly in case of non-mass-like enhancements, where lesion distribution is diffuse and interspersed with normal fibroglandular and adipose tissue, clinical palpation may not always detect a distinct mass, thus complicating surgical diagnostics. Conversely, advanced deep learning methods can accurately delineate clinical lesion size, volume, and characteristics while identifying subtle image feature variations that may be overlooked by manual methods.

The effectiveness of the lightweight MM-3DUNet for breast cancer mass segmentation has been validated across various medical segmentation domains. For example, Yu et al. (28) applied depth-wise separable convolutions for glioma imaging segmentation, resulting in a significant reduction in model computation time. Ma et al. (29) on the other hand, combined multilayer perceptrons with depth-wise separable convolutions to create LMU-Net, a lightweight medical imaging segmentation model which reduced computational demands by nearly 50% compared to baseline models. By incorporating this strategy into breast cancer mass segmentation, the training process is accelerated and enables deployment in resource-constrained environments.

However, constructing such networks still confronts the inherent challenge of balancing higher accuracy with increased computational demands. Although MM-3DUNet reduces parameter counts and computational needs through its lightweight design, this simplification might potentially compromise segmentation accuracy in certain cases. To address this concern, we have implemented a series of strategies to balance accuracy and efficiency, including adjustable channel expansion factors in the CEC unit to balance computational complexity and segmentation accuracy (30, 31). While most lightweight breast cancer segmentation networks prefer 2D slices over 3D imaging, our model adjusts parameters flexibly based on specific dataset and task requirements to meet diverse clinical application needs. Additionally, this study innovatively includes ACT at the network’s end, a strategy whose effectiveness has been amply validated in various medical imaging scenarios such as ventricular, cerebral, and hepatic vessel segmentation (32, 33).

This research repurposes learned features to determine the presence of lesion information in input images, providing supervision for the primary segmentation task to avoid loss of crucial information in deep learning and improve segmentation accuracy for small area targets. However, the study still faces limitations, as single input and convolution operations struggle with real-time and generalization capabilities in large-scale or diverse clinical datasets. Future research will aim to expand data collection, optimize 3D convolutional structures, and explore integration with other advanced deep learning frameworks such as Transformers to enhance the potential of multimodal inputs in lightweight medical segmentation scenarios. This will further improve computational efficiency and segmentation performance while enhancing network stability and adaptability.

## 5 Conclusion

Addressing the challenges posed by high parameter count and computational demands associated with the classical 3DUNet network for MRI segmentation, this study introduces MM-3DUNet, a lightweight segmentation network enhanced with multi-task depth-wise separable convolution. This innovative network achieves a notable reduction in both model parameters and computational complexity while enhancing the segmentation accuracy for breast cancer tumor masses. Initially, depth-wise separable convolutions were applied to the convolutional computations of 3DUNet, supplemented by CEC unit and IRB. These modifications not only streamline the network’s efficiency but also bolster its feature extraction capabilities, leading to improved segmentation performance. Furthermore, ACT was incorporated during the decoding phase of the network to provide additional supervisory signals for the primary segmentation task, further enhancing the segmentation accuracy of breast cancer masses under reduced computational complexity. Experimental results demonstrate that compared to the classical 3DUNet, MM-3DUNet achieves a remarkable reduction of 63.16% in parameters and 80.90% in FLOPs, substantially lowering the model’s computational complexity. In terms of segmentation accuracy, MM-3DUNet shows an increase of 1.30% in IoU compared to 3DUNet, thus achieving a lighter model while enhancing the precision of breast cancer mass segmentation.

## Data Availability

The existing dataset will be made public. Requests to access these datasets should be directed to wangxy198812@163.com.
